# Supplementary material for: Multidimensional performance characteristics of youth academy and club soccer players
Source: PLoS One. 2026 May 15;21(5):e0348716. doi: 10.1371/journal.pone.0348716 (PMC13178925; doi:10.1371/journal.pone.0348716)
Supplement: S1 Table — (DOCX) [file pone.0348716.s001.docx]

# Supporting information

S1 File.

**Table S1.** Cognitive outcome measures

|  | Outcome Measure Code | Description | Cognitive Domain |
| --- | --- | --- | --- |
| RTI |  |  |  |
|  | RTIFMDMT | Motor response time milliseconds (five-choice) | Processing speed |
|  | RTIFMDRT | Reaction time milliseconds (five-choice) | Processing speed / Attention |
| SSP |  |  |  |
|  | SSPFSL | Maximum Span Length reached | Working memory (visuospatial) |
| SST |  |  |  |
|  | SSTSSRT | Stop-signal reaction time | Inhibitory control / Response inhibition |
| IED |  |  |  |
|  | IEDEEDS* | Errors during extra-dimensional shifts | Cognitive flexibility / Set shifting |
|  | IEDYERTA | Total errors (adjusted for stages reached) | Cognitive flexibility / Error monitoring |
| CGT |  |  |  |
|  | CGTDAVT | Delay aversion total (risk-taking differences between conditions) | Decision making / Impulsivity |
|  | CGTRAJTM | Risk adjustment merged (sensitivity to outcome probability) | Decision making / Rational thinking |
|  | CGTRTKMT | Risk adjustment total merged (risks on high probability trial) | Decision making / processing speed |

Note. Outcome measures were derived from the Cambridge Neuropsychological Test Automated Battery (CANTAB). Abbreviations: RTI= Reaction time; SSP; Spatial Span; SST= Stop Signal Task; IED= Intra-Extra Dimensional Set Shift; CGT= Cambridge Gambling Task. Each outcome measure is associated with a particular cognitive domain.
