# Supplementary material for: Multidimensional performance characteristics of youth academy and club soccer players
Source: PLoS One. 2026 May 15;21(5):e0348716. doi: 10.1371/journal.pone.0348716 (PMC13178925; doi:10.1371/journal.pone.0348716)
Supplement: S2 Table — (DOCX) [file pone.0348716.s002.docx]

# S2 Table. Reliability and validity of measurement instruments used in the study

| **Test** | **Domain** | **Validity** | **Reliability** | **Key references** |
| --- | --- | --- | --- | --- |
| Bone age (X‑ray, BoneXpert) | Skeletal age | Considered gold standard for assessing skeletal age in youth, high criterion validity | High reliability: automated scoring reduces inter- and intra-rater error | (49) |
| Height and weight (standardized stadiometer & scale) | Anthropometrics | High face and criterion validity | High test- retest reliability when using standardized protocol |  |
| 40 m linear sprint | Physical performance - speed | Valid measure of linear sprint ability; discriminates between performance levels | High reliability (ICC = 0.87-0.99) | (20) |
| Yo‑Yo Intermittent Recovery Test Level 1 (IR1) | Aerobic capacity / intermittent endurance | Strong construct validity for youth soccer; reflects match‑related demands | High reliability (ICC ≈ 0.87–0.95) | (53) |
| MST‑f (Mental Skills Test – Football) | Psychological skills | Exploratory factor analysis indicates acceptable validity; football‑specific design increases ecological validity | Satisfactory reliability reported in validation work | (56, 59) |
| BRSQ‑20 (motivation) | Motivation (SDT framework) | Validated in youth populations and across European languages | Good internal consistency (α typically 0.70–0.90) | (60,62) |
| CANTAB Stop Signal Task (SST) | Inhibition | Valid measure of response inhibition; sensitive to developmental differences | ICC ≈ 0.71–0.72 | (72-75) |
| CANTAB Spatial Span (SSP) | Working memory | Strong construct validity; digital version of validated Corsi Block Test | ICC ≈ 0.51 (children), 0.64 (adults) | (76,87,103,77) |
| CANTAB Intra‑Extra Dimensional Set Shift (IED) | Cognitive flexibility | Digital analogue of Wisconsin Card Sorting Test; moderate validity | Test–retest correlations r ≈ .38–.78 | (71,78,79) |
| CANTAB Reaction Time (RTI) | Psychomotor speed / attention | Moderate correlations with other RT measures | Test–retest r ≈ .55–.84 | (80-82) |
| CANTAB Cambridge Gambling Task (CGT) | Decision‑making / risk assessment | fMRI studies support neural validity; limited youth‑specific validation | Limited reliability data in youth; acceptable in adults | (83) |
