# Supplementary material for: Multidimensional performance characteristics of youth academy and club soccer players
Source: PLoS One. 2026 May 15;21(5):e0348716. doi: 10.1371/journal.pone.0348716 (PMC13178925; doi:10.1371/journal.pone.0348716)
Supplement: Attachment — Submitted filename: Response to Reviewers-Plos One.docx [file pone.0348716.s004.docx]

**Response to Reviewers – PLOS ONE**

**Manuscript title:** *Multidimensional Performance Characteristics of Youth Academy and Club Soccer Players* **Manuscript ID:** PONE-D-25-64451 **Journal:** PLOS ONE

**Dear Academic Editor and Reviewers,**

We would like to thank you for the thorough and constructive feedback on our manuscript. We appreciate the time and expertise invested in reviewing our work. We have carefully revised the manuscript in accordance with all comments and suggestions. Below, we provide a detailed, point‑by‑point response. All changes are marked in the revised manuscript with track changes.

**Journal Requirements Response**

*1.In the online submission form you indicate that your data is not available for proprietary reasons and have provided a contact point for accessing this data. Please note that your current contact point is a co-author on this manuscript. According to our Data Policy, the contact point must not be an author on the manuscript and must be an institutional contact, ideally not an individual. Please revise your data statement to a non-author institutional point of contact, such as a data access or ethics committee, and send this to us via return email. Please also include contact information for the third party organization, and please include the full citation of where the data can be found.*

**Thank you for your message and the opportunity to clarify the data availability conditions for our manuscript. Below we address all requested points, including revisions to the data access contact and an explanation of the ethical and legal restrictions that prevent public sharing of the dataset***.***Please note that the corresponding author for this manuscript will be Morten Kristoffersen, not Ulrikke Norill Kvalvaag.**

**Correction regarding institutional approval (Sikt)
The project underwent a formal data protection assessment conducted by Sikt – Norwegian Agency for Shared Services in Education and Research. Sikt performs a data protection assessment on behalf of our institution (Western Norway University of Applied Sciences, HVL), while all responsibility for data processing and data protection compliance remains with HVL.**

**Revision of data access contact (non-author, institutional)
We acknowledge your requirement that the data access contact must not be an author on the manuscript and must be an institutional contact point.

Institutional Data Access Contact (non-author):
Data Protection Officer (Personvernombud)
Western Norway University of Applied Sciences (HVL)
Email: personvern-afii@hvl.no**

**This institutional office is responsible for evaluating any data requests under applicable regulations. The data are securely stored by HVL and not held by a third-party organization. No repository citation can be provided because the dataset cannot be publicly deposited due to the ethical and legal constraints outlined below.**

*2.We note that you have indicated that there are restrictions to data sharing for this study. PLOS only allows data to be available upon request if there are legal or ethical restrictions on sharing data publicly. For more information on unacceptable data access restrictions, please see* [*http://journals.plos.org/plosone/s/data-availability#loc-unacceptable-data-access-restrictions*](http://journals.plos.org/plosone/s/data-availability#loc-unacceptable-data-access-restrictions)*.*

***Before we proceed with your manuscript, please address the following prompts:***

*a) If there are ethical or legal restrictions on sharing a de-identified data set, please explain them in detail (e.g., data contain potentially identifying or sensitive patient information, data are owned by a third-party organization, etc.) and who has imposed them (e.g., a Research Ethics Committee or Institutional Review Board, etc.). Please also provide contact information for a data access committee, ethics committee, or other institutional body to which data requests may be sent.*

**Ethical and legal restrictions on data sharing
 a) Explanation of restrictions, who imposed them, and why the dataset cannot be shared:
There are legal and ethical restrictions that prevent public sharing of the dataset, even in de-identified form. The study involves minors (approximately 14 years old), who are considered a vulnerable population under Norwegian and EU data protection regulations (GDPR). Although direct identifiers were never included in the research dataset, the combination of several variables (age, sport-specific performance metrics, school/sports affiliation, training history, and other contextual variables) creates a significant risk of backdoor re-identification. This risk was explicitly raised during a consultation with Sikt on 20 February 2026 and was assessed as non-negligible due to the small and specific recruitment population.

As a result:
- Sikt’s data protection assessment concluded that the dataset cannot be sufficiently anonymized without compromising data integrity and leaving a residual re-identification risk.
- HVL’s Data Protection Officer imposed restrictions preventing any public sharing of the dataset, including deposition in open repositories.
- These restrictions fall under the Norwegian Personal Data Act and GDPR, which prohibit sharing data that cannot be rendered fully anonymous.

Institutional contact for data inquiries:
Data Protection Officer (Personvernombud)
Western Norway University of Applied Sciences (HVL)
Email: personvern-afii@hvl.no
Phone: +47 55587682

Data may be shared only under controlled conditions and only after independent evaluation ensuring full compliance with ethical and legal requirements. Open or public access is not permitted.**
 *b) If there are no restrictions, please upload the minimal anonymized data set necessary to replicate your study findings to a stable, public repository and provide us with the relevant URLs, DOIs, or accession numbers. For a list of recommended repositories, please see*
[*https://journals.plos.org/plosone/s/recommended-repositories*](https://journals.plos.org/plosone/s/recommended-repositories)*. You also have the option of uploading the data as Supporting Information files, but we would recommend depositing data directly to a data repository if possible.*

**We will update your Data Availability statement on your behalf to reflect the information you provide**.

**b) Upload of anonymized data (not applicable)
It is not legally or ethically permissible to upload a de-identified dataset to a public repository due to the risk of re-identification and because the participants are minors. The restrictions were imposed by HVL’s Data Protection Officer following the official data protection assessment. Accordingly, option (b) does not apply.

Revised Data Availability Statement (for the manuscript)
Data Availability Statement:
The data from this study involve minors and contain sensitive information that cannot be publicly shared due to ethical and legal restrictions imposed by the institutional Data Protection Officer at Western Norway University of Applied Sciences (HVL), following a formal data protection assessment by Sikt – Norwegian Agency for Shared Services in Education and Research. Due to the small and specific population studied, even de-identified data carry a risk of participant re-identification. Data may be made available upon reasonable request to the HVL Data Protection Officer (contact information above), subject to approval under applicable ethical and data protection regulations. Public access or repository storage is not permitted.

Please let us know if any further clarifications or revisions are needed.**

**Response to Reviewer #1**

**General comment**

We thank the reviewer for the positive evaluation of the study and for the helpful suggestions. We have addressed all points as detailed below.

**1. Hypotheses (clarity and presentation)**

**Reviewer comment:** *The hypotheses should be stated more explicitly. Please present the study hypotheses in a clear, direct format (ideally numbered or listed) so the reader can easily understand what was tested and how the analyses relate to the research aims*.

**Response:** Thank you for this suggestion. We have now added a clearly structured, numbered list of hypotheses at the end of the Introduction. These hypotheses directly reflect the study aims. (P.6, Line 199-203)

**2. Reliability and validity reporting**

**Reviewer comment:** *While the selected tests and measurements appear appropriate, the manuscript needs clearer reporting of reliability and validity for all instruments and assessments used. Please add a concise section (or table) summarizing reliability/validity evidence for each test/measurement (e.g., test–retest reliability, inter-rater reliability if applicable, internal consistency when relevant, and validity evidence). If reliability was assessed in the current sample, those results should be reported; if not, provide strong supporting citations and justify why those measures are appropriate for the population and context.*

**Response:** We agree and have now added a dedicated supporting file titled “S2 Table. Reliability and Validity of Measurement instrument used in the study”. Relevant citations have been added. We have also reported internal consistency for the questionaries in mental skills and motivation under the method section (P10, line- 296-301) and (P.11, line 317-319)

**3. Literature review (update and scope)**

**Reviewer comment: *Literature review (update and scope)
o The discussion and conclusion would benefit from incorporating more recent literature (2023 and above) to reflect current debates and developments in soccer talent identification.
o In addition, I recommend strengthening the literature review by including a focused section on key problems/challenges in talent identification in football, such as (as relevant to the manuscript): relative age effects, maturation status and growth-related bias, contextual and environmental influences, position-specific demands, selection biases, and the limitations of predicting long-term performance from early testing*.**

**Response:** Thank you, we agree and have revised the discussion and conclusion. In addition, we have strengthened the introduction and included more of the key challenges in talent identification. We have included the following references:

Shahidi, S. H., Carlberg, B., & J, D. K. (2023). Talent Identification and Development in Youth Sports: A Systematic Review. *International Journal of Kinanthropometry*, *3*(1), 73–84. <https://doi.org/10.34256/ijk2318>

Martinho, D. V., Gonzalo-Skok, O., Chamari, K., Field, A., Clemente, F. M., & Rebelo, A. et al. (2026). Nutrition as a missing piece in the development of youth male soccer players: a scoping review and future directions. Biology of Sport, 43(1), 291-317.

Martinho, D. V., Coelho-E-Silva, M. J., Gonçalves Santos, J., Oliveira, T. G., Minderico, C. S., Seabra, A., Valente-Dos-Santos, J., Sherar, L. B., & Malina, R. M. (2023). Body Size, Fatness and Skeletal Age in Female Youth Soccer Players. International Journal of Sports Medicine, 44(10), 711–719. <https://doi.org/10.1055/a-1686-4563>

Till K and Baker J (2020) Challenges and [Possible] Solutions to Optimizing Talent Identification and Development in Sport. Front. Psychol. 11:664. doi: 10.3389/fpsyg.2020.00664 (nummer 10)

Maratova K, Zemkova D, Sedlak P, Pavlikova M, Amaratunga SA, Krasnicanova H, Soucek O, Sumnik Z. A comprehensive validation study of the latest version of BoneXpert on a large cohort of Caucasian children and adolescents. Front Endocrinol (Lausanne). 2023 Mar 24;14:1130580. doi: 10.3389/fendo.2023.1130580. PMID: 37033216; PMCID: PMC10079872.

**4. Discussion and conclusion (strength, practical applications, future directions)**

**Reviewer comment*:*** *The conclusion section should be strengthened to provide clearer practical applications for coaches, academies, and practitioners, aligned with the study findings. Please also expand the future research directions, including what additional variables, methods, samples, or longitudinal approaches might address limitations in the current study and improve talent identification models.*

**Response:** Thank you for the comment. The conclusion has been revised to include clearer practical applications and more specific future research directions (P.24, line 652- 686)

**Response to Reviewer #2**

**General comment**

We thank the reviewer for the constructive and detailed feedback. All points have been addressed as outlined below.

**[1] Clarification of early talent detection**

**Reviewer comment:** *P3 L36-38: The idea presented contradicts the initial assertion. You state, “First, to ensure that the most promising talent reaches their full potential, clubs and national federations allocate substantial resources to identifying and nurturing players as early as possible.” If the dynamics of sports change in adulthood, why is there an emphasis on early identification?*

**Response:** We agree that the original phrasing did not sufficiently clarify that early identification is a common *practice* in clubs and federations, but not necessarily an *evidence‑based* or effective strategy. We have now revised the introduction to make this explicit. The new wording explains the difference between identification, development and selection. And further explains how early selection occurs largely due to structural and competitive pressures in talent‑development systems, rather than because early performance is a reliable predictor of long‑term success. (P3. lines 40–50)

**[2] Definition of “early talent detection”**

**Reviewer comment:** Please clarify the term "early talent detection." What does it specifically entail?

**Response:** Thank you for this comment. We agree that term *“early talent detection.”* Was not appropriately used in the sentence*.* However, this specific term is **not used or defined** in our study, as our focus is solely on **talent identification, talent development, and talent selection**. We have revised the relevant sentence in the introduction so that it no longer use of the term “early talent detection.” The definitions and terms is now updated. (p. 3, lines 40–50).

**[3] Additional factors influencing coach selection**

**Reviewer comment:** P3 L42: While the authors address maturation and the relative age effect, it is important to recognize that other factors influence coach selection. Speculation should be balanced with these additional considerations.

**Response:** We thank the reviewer for this important comment. The introduction has been revised to acknowledge that coach selection decisions are influenced by a broader range of factors beyond biological maturation and relative age effects. Specifically, we added how coach perceptions of talent, as well objective testing may influence selection decisions within talent identification systems. The relevant changes have been incorporated in (Page 4, lines 85-89).

**[4] Add recent references**

**Reviewer comment** P3 L33-P42: Recent references relevant to youth soccer players could enhance the introduction section. Consider including the following:
Martinho, D. V., Gonzalo-Skok, O., Chamari, K., Field, A., Clemente, F. M., & Rebelo, A. et al. (2026). Nutrition as a missing piece in the development of youth male soccer players: a scoping review and future directions. Biology of Sport, 43(1), 291-317.
Martinho, D. V., Coelho-E-Silva, M. J., Gonçalves Santos, J., Oliveira, T. G., Minderico, C. S., Seabra, A., Valente-Dos-Santos, J., Sherar, L. B., & Malina, R. M. (2023). Body Size, Fatness and Skeletal Age in Female Youth Soccer Players. International Journal of Sports Medicine, 44(10), 711–719. <https://doi.org/10.1055/a-1686-4563>

**Response:** Thank you for the suggestion. The references has been added: Martinho 2023- (added in page 4 line 93), Marthino 2026, (added in page 8, line 259)

**[5] Terminology: “skeletal age”**

**Reviewer comment**: P3 L49: It may be more accurate to refer to "skeletal age" rather than "bone age."

**Response:** In response to the reviewer’s comment, the term bone age has been replaced with skeletal age throughout the manuscript.

**[6] Incorrect use of “physiological development”**

**Reviewer comment**: P3 L50: The term "physiological development" is incorrect; please revise accordingly.

**Response:** We have revised the sentence to use the term **“biological maturation,”** which better reflects what bone age estimation is intended to represent.

**[7] Consistent terminology (identification/selection/development)**

**Reviewer comment**: P4 L55-58: Ensure consistent terminology throughout the text. "Identification," "selection," and "development" represent different concepts. Refer to the following sources for clarity:
Till, K., & Baker, J. (2020). Challenges and [Possible] Solutions to Optimizing Talent Identification and Development in Sport. Frontiers in Psychology, 11, 664. <https://doi.org/10.3389/fpsyg.2020.00664>
Mendes, D., Martinho, D. V., Travassos, B., Gouveia, É. R., Saavedra, N. O., & Sarmento, H. (2025). Talent Selection in Portuguese National Futsal Teams. Journal of Human Kinetics, 99, 253–261. <https://doi.org/10.5114/jhk/199379>

**Response:** We have revised the Introduction to ensure consistent use of terminology, supported by the recommended references (Till and baker 2020), (P3, line 40-50).

**[8] Introduction length**

**Reviewer comment**: The introduction is overly lengthy. Consider abbreviating the last two paragraphs leading up to the study's aim

**Response:** Thank you for this comment. We revised the final two paragraphs leading up to the study aim to improve clarity and focus. However, based on other reviewer comments, additional clarifications and references were incorporated elsewhere in the introduction. As a result, the overall length was not substantially reduced, but the structure and clarity of the introduction have been improved.

**[9] Categorization of maturational status**

**Reviewer comment**: P7 L142: How do you categorize players by maturational status? The authors represent skeletal age as a continuous variable, rather than categorical.

**Response:** Thank you for the comment. In our study, skeletal age was intentionally treated as a continuous indicator of biological maturation, and we did not categorize players into maturational groups (e.g., early/on‑time/late). Categorizing skeletal age requires arbitrary cut‑offs and reduces sensitivity and statistical power. Because our analyses examine maturation as a continuous developmental process, modelling skeletal age continuously provides a more precise and methodologically robust representation of maturational status. We have now clarified this in the Methods section. (P.8. 246-248).

**[10] Data quality for skeletal age assessment**

**Reviewer comment**: Please discuss the data quality related to skeletal age assessment, including citations from other studies to support your findings

**Response:** Thank you for this comment. We have expanded the manuscript to further discuss the data quality of the skeletal age assessment. Specifically, we added information about the reported accuracy of the BoneXpert method, which has demonstrated good agreement with manual bone age assessment with a root mean square error (RMSE) of approximately 0.68 years in males and 0.52 years in females. We also acknowledge that skeletal age estimates can vary depending on the assessment protocol used, emphasizing the importance of methodological consistency when interpreting biological maturation. This information has now been added to the Methods section (Page 8, Line 256-259).

**[11] Greulich–Pyle variability**

**Reviewer comment**: P8 L149-150: The reduction of variability in the Greulich-Pyle method has been noted in female soccer, as documented in the following study:
Martinho, D. V., Coelho-E-Silva, M. J., Valente-Dos-Santos, J., Minderico, C., Oliveira, T. G., Rodrigues, I., Conde, J., Sherar, L. B., & Malina, R. M. (2022). Assessment of skeletal age in youth female soccer players: Agreement between Greulich-Pyle and Fels protocols. American Journal of Human Biology, 34(1), e23591. <https://doi.org/10.1002/ajhb.23591>

**Response:** Thank you for this comment. In line with question 10, we have added recommended reference to acknowledge that skeletal age estimates may vary depending on the assessment protocol applied. In the present study, skeletal age was assessed using a single standardized protocol implemented through automated BoneXpert analysis, ensuring methodological consistency across all participants. (Page 8, Line 256-259).

**[12] Table labels**

**Reviewer comment**: Tables should not use labels A, B, and C. Please replace these with "academy players," "non-match academy players," and "club players."

**Response:** Table labels A/B/C have been replaced with the descriptive group names: Match-selected academy players, non-match academy players and club players. Post-hoc directions has been switched to MS (Match-selected), NMS (Non-match-selected), CP (Club-players).

**[13] Figures and supplementary material**

**Reviewer comment**: Consider adding some tables as supplementary material and designing figures for the main results to present more objective findings.

**Response:** Thank you for this suggestion. However, we do not think we have additional data that are relevant as supplementary data in this article. We also believe the tables give the best presentation of our data. However, if you have specific suggestions as to what data may be presented as figures we are happy to reconsider.

**Closing statement**

We hope that the revisions meet the expectations of the reviewers and the editor. We appreciate the opportunity to improve our manuscript and look forward to your further consideration.

**Sincerely,**

Ulrikke Norill Kvalvaag
On behalf of all authors
